# A comparison between laterally wedged insoles and ankle-foot orthoses for the treatment of medial osteoarthritis of the knee: A randomized cross-over trial

**DOI:** 10.1177/0269215521993636

**Published:** 2021-03-29

**Authors:** Martin Schwarze, Leonie P Bartsch, Julia Block, Merkur Alimusaj, Ayham Jaber, Marcus Schiltenwolf, Sebastian I Wolf

**Affiliations:** Department of Orthopedics, University Hospital Heidelberg, Heidelberg, Germany

**Keywords:** Ankle-foot orthosis, insoles, knee osteoarthritis, orthopedic aid, health-related quality of life

## Abstract

**Objective:**

To compare biomechanical and clinical outcome of laterally wedged insoles (LWI) and an ankle-foot orthosis (AFO) in patients with medial knee osteoarthritis.

**Design:**

Single-centre, block-randomized, cross-over controlled trial.

**Setting:**

Outpatient clinic.

**Subjects:**

About 39 patients with symptomatic medial knee osteoarthritis.

**Interventions:**

Patients started with either LWI or AFO, determined randomly, and six weeks later changed to the alternative.

**Main measures:**

Change in the 1st maximum of external knee adduction moment (eKAM) was assessed with gait analysis. Additional outcomes were other kinetic and kinematic changes and the patient-reported outcomes EQ-5D-5L, Oxford Knee Score (OKS), American Knee Society Clinical Rating System (AKSS), Hannover Functional Ability Questionnaire – Osteoarthritis and knee pain.

**Results:**

Mean age (SD) of the study population was 58 (8) years, mean BMI 30 (5). Both aids significantly improved OKS (LWI *P* = 0.003, AFO *P* = 0.001), AKSS Knee Score (LWI *P* = 0.01, AFO *P* = 0.004) and EQ-5D-5L Index (LWI *P* = 0.001, AFO *P* = 0.002). AFO reduced the 1st maximum of eKAM by 18% (*P* < 0.001). The LWI reduced both maxima by 6% (*P* = 0.02, *P* = 0.03). Both AFO and LWI reduced the knee adduction angular impulse (KAAI) by 11% (*P* < 0.001) and 5% (*P* = 0.05) respectively. The eKAM (1st maximum) and KAAI reduction was significantly larger with AFO than with LWI (*P* = 0.001, *P* = 0.004).

**Conclusions:**

AFO reduces medial knee load more than LWI. Nevertheless, no clinical superiority of either of the two aids could be shown.

## Introduction

Lateral wedge insoles and ankle-foot orthoses are therapeutic options in mild to moderate medial knee osteoarthritis.^1,2^ Both devices aim for medial compartment relief to prevent increased loading of the medial joint compartment during gait and negate this effect for the initiation and progression of osteoarthritis.^3,4^ The knee adduction moment is a commonly used and validated surrogate parameter for medial knee load in the stance phase of gait.^5^ Its reduction is intended to reduce pain, improve joint function and slow down disease progression.^6^ Assuming a comparable indication and biomechanical concept of both lateral wedge insoles and ankle-foot orthosis, the question remains unanswered whether one of the two aids should be preferred.

Few studies directly compare lateral wedge insoles with ankle-foot orthoses. In a healthy study population, the knee adduction moment was reduced more effectively by the orthosis than by a lateral wedge insole.^7^ Although this difference was reproduced in a population with knee osteoarthritis,^8^ both studies worked with a one-day laboratory assessment.

To date, no clinical trial directly compares the mid-term effects of ankle-foot orthosis and lateral wedge insole in patients with mild to moderate medial knee osteoarthritis. The aim of this study was therefore to compare both biomechanical and clinical effects of a six-week intervention with each device in a population with medial knee osteoarthritis.

Primarily, the reduction of medial knee load by each aid would be examined and compared, as this is the targeted mechanism of action of both aids. A range of patient-reported outcome measures for knee pain, knee function and health-related quality of life were evaluated to allow application in the clinical setting.

## Patients and methods

This was a single-centre cross-over study conducted at the Heidelberg University Hospital, Clinic for Orthopedics and Trauma Surgery. Data acquisition and analysis were performed in compliance with protocols approved by the Ethical Committee of the medical faculty of the Ruprecht Karl University of Heidelberg (S-021/2018). The study was registered in the German Register of Clinical Studies (DRKS00016783). The study was conducted in accordance with the Declaration of Helsinki. Financial support was given by “Deutsche Arthrose-Hilfe e.V.” with 12.000 €. Remaining costs were covered by research funds of the Orthopedic Clinic of Heidelberg University Hospital.

Patients with symptomatic medial knee osteoarthritis were recruited from the outpatient orthopedic clinic of Heidelberg University Hospital. Over a period of 16 months (05/2018-08/2019), 42 participants were included. All participants provided written informed consent. Inclusion criteria included the following:

- Age: At least 18 years of age
- Knee osteoarthritis stage 1–3 according to Kellgren and Lawrence^9^
- No previous orthopedic (shoe) technical treatment due to knee osteoarthritis
- No previous operations due to knee osteoarthritis
- Ability to walk

In a cross-over design, each patient received both interventions for six weeks each in a randomized order. A block-randomization process with variable block size (up to 16) was used to determine which treatment, insole or orthosis, was used for the first six weeks. If both knees met the inclusion criteria, both knees were treated simultaneously with the same intervention.

The alternate was used over the second six weeks. The treatment sequence was assigned with a balanced, block-wisely randomized allocation list in MatLab (The Math Works, Inc.). The allocation was known to patients and researchers.

**Figure 1.**
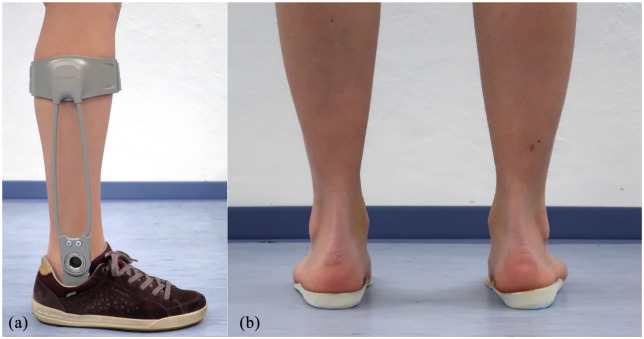
(a) Agilium Freestep 2.0 ankle-foot orthosis, and (b) insole with 5 mm lateral wedge on the right, neutral insole on the left side.

The specific treatments were:

1. Agilium Freestep *orthosis* (Figure 1(a), Otto Bock HealthCare, Duderstadt, Germany): Its semi-rigid footplate is adapted to the patient’s own footwear. The talotarsal joint is bridged via a hinged joint with free motion in the sagittal plane and subtotal restriction in the frontal and transverse plane. There is a connection to the flat lateral shank inlay, which is adjusted to the individual anatomy according to the manufacturer’s instructions. Supplied in combination with neutral soft foam insoles with longitudinal and transverse arch support (Hepuflex Business Mikrofaser, Art.-Nr. 5513-022, HEMA Orthopädische Systeme GmbH, Tunzenhausen, Germany).
2. Long-sole *insoles* with longitudinal and transverse arch support with high-shore 5 mm lateral wedge (Figure 1(b)) based on the initial description by Sasaki and Yasuda.^10,11^

The supply of aids, adaptation and training in use were carried out by the Department of Technical Orthopedics at Heidelberg University Hospital (certified according to ISO 9001 and 13485). After adaptation of the first aid, the participants were instructed to use it in everyday life for a period of six weeks before switching to the other aid for another six weeks. After completing the 12-week study protocol, the participants were left with the option to continue using any one of the two aids tested.

Patients underwent three examinations: At baseline, after six weeks (first intervention) and after 12 weeks (second intervention). The primary outcome measure was the change in knee adduction moment with each of the orthopedic aids compared to the baseline. Secondary outcome measures were changes in patient-reported outcomes and in other gait analysis parameters. Additionally, a standardized clinical examination was conducted at baseline.

In case of bilateral use of the intervention, only the more symptomatic knee of the patient was included in the data analysis. In case of bilaterally equal symptoms and equal radiographic severity on both sides, the assessed side was randomized by a coin toss (one case).

The baseline clinical examination included the passive range of motion of hip, knee and ankle joints. The following foot deformities were assessed on a semi-quantitative scale (none – slight – marked): Pes equinus, pes valgus, pes varus, flat foot, pes adductus, pes excavatus, hallux valgus.

To evaluate clinical effects of the aids, various patient-reported outcome measures were assessed at each examination date:

- EQ-5D-5L.^12^
- Oxford Knee Score:^13^ 12-item questionnaire for patients with knee replacement assessing pain and subjective functional limitation of the knee for the last four weeks, scores ranging from 0 (worst) to 48 points (best).
- American Knee Society Clinical Rating System:^14^ Multidimensional questionnaire including the cause of impairment (unilateral/bilateral knee disease/other disease), a subjective “Function Score” and a “Knee Score” based on repeated clinical examination, each reaching a maximum of 100 points.
- Hannover Functional Ability Questionnaire – Osteoarthritis:^15^ Functional questionnaire about everyday activities. Values ⩾ 80% correspond to a normal finding and <60% to a clinically relevant functional impairment.
- Knee Pain: Knee pain on the affected side in the last seven days was documented on a numerical rating scale from 0 (no pain) to 10 (worst imaginable pain).

To evaluate biomechanical effects of the aids, instrumented 3D gait analysis was carried out at baseline and after each six-week intervention period.

For the gait analysis, a 120 Hz 12-camera system (Vicon, Oxford, United Kingdom) and two force plates (Kistler, Winterthur, Switzerland) were used. Reflective markers were placed on bony landmarks according to PlugIn Gait (Vicon, Oxford, United Kingdom.), based on the models of the lower extremity according to Kadaba et al.^16^ and Davis et al.^17^ While walking with the ankle-foot orthosis, no marker could be attached to the lateral malleolus on the affected side. The marker was placed on the orthosis instead. The true position of the lateral malleolus was defined in relation to four additional cluster markers on the frontal tibia in a static calibration trial.^18^

**Figure 2.**
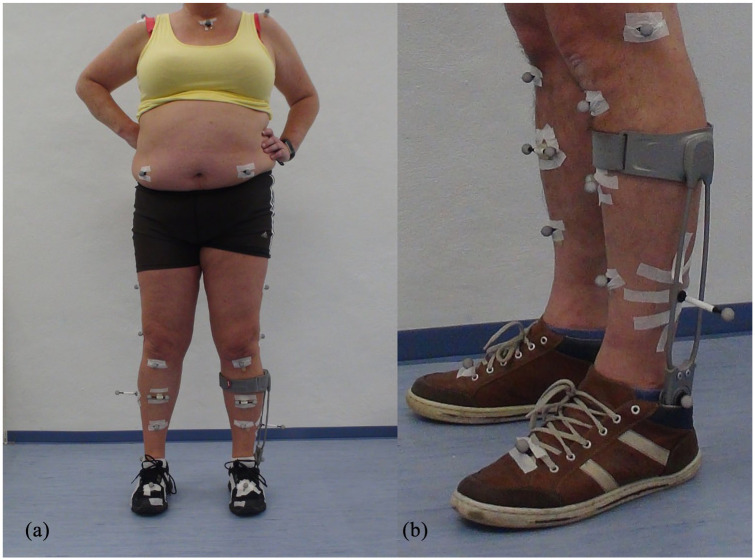
(a) Modified PlugInGait marker setup with 4 additional markers on the tibial tuberosity and tibial crest, and (b) The left ankle marker is placed in the sagittal joint of the ankle-foot orthosis (different patient). Its position is later replaced by the ankle position relative to the four tibial markers.

Figure 2 illustrates the marker setup.

The patients walked a distance of 7 m at a self-selected speed. The footwear was identical at all examination dates. After each intervention period, the corresponding aid was worn in the gait analysis. Motion data was recorded using the software Vicon Nexus 2.8 (Vicon, Oxford, United Kingdom). A minimum of eight trials per walking condition were averaged.

The evaluation of the gait analysis was focused on:

- External knee adduction moment (primary endpoint)
- Knee adduction angular impulse (integral of the external knee adduction moment that represents its cumulative effect over the stance phase of gait)
- External knee flexion moment
- Temporo-spatial parameters

## Statistical analysis

The statistical evaluation was carried out after qualified statistical advice. The case number planning was calculated for the primary outcome measure and the reduction of peak knee adduction moment compared to baseline. Based on a study by Fantini Pagani et al.^7^ who found a reduction of 12% with the ankle-foot orthosis (effect size of 0.541), the case number was calculated using a *t*-test based model in G * Power version 3.1.5.^19^ A power of 0.95 with a significance level of 0.05 was chosen and resulted in a required number of 47 cases. A post-hoc power analysis was carried out with the same software.

As primary endpoint, the 1st maximum of the external knee adduction moment of each orthopedic aid was compared to the baseline separately. After verifying normal distribution, a two-sided t-test for paired samples was used. In case of non-normal distribution, a Wilcoxon signed-rank test was used. It was defined as the null hypothesis that the first maximum of the external knee adduction moment in the gait cycle does not differ between gait analysis with shoes only and gait analysis with additional ankle-foot orthosis or lateral wedge insole.

The anthropometric data as well as age, gender distribution and degree of knee osteoarthritis were evaluated with an unpaired *t*-test or a Mann-Whitney *U* test for non-interval-scaled variables.

The clinical scores and other gait analysis parameters were evaluated descriptively. Mean values, standard deviation, minimum, median, maximum and descriptive *P*-values (paired t-test in each case compared to the baseline) were used for continuous parameters. The absolute and relative frequencies were considered for categorical data. After showing a lack of normal distribution in the Shapiro-Wilk test, the statistical evaluation of all patient-reported outcome measures was carried out using the Wilcoxon signed-rank test.

For direct comparison of outcome parameters between the two interventions, a crossover analysis was used as described by Wellek and Blettner^20^ In order to test the influence of period effects on test results, Wellek and Blettner recommend a t-test for independent samples between treatment sequence cohorts. Additionally, a two-step analysis is recommended to verify the absence of treatment-dependent carryover effects before comparing the actual treatment effects.

In a first step, global differences of outcome measures depending on the treatment sequence were assessed. Intraindividual sums of outcome parameters at six and twelve weeks were calculated. Normal distribution of the sums was verified. A two-sided unpaired *t*-test was then used to compare the intraindividual sums between the two treatment sequence cohorts. For ordinally scaled variables or if normal distribution was not given, a Mann-Whitney-*U*-test was used instead. This pre-test was not significant at a level of 0.05 for any of the parameters tested. As a result, we assumed no carryover effect and proceeded with the second step of analysis.

In the second step, effects of the two treatments were compared with each other. Intraindividual differences of twelve minus six weeks were calculated for outcome parameters. A two-sided unpaired *t*-test was used to compare intraindividual differences between treatment sequence cohorts: ankle-foot orthosis first and lateral wedge insole first. In case of ordinally-scaled variables or a non-normal distribution of the intraindividual differences in each sequence group, a Mann-Whitney-*U*-test was used instead. *P*-values obtained in this testing procedure are solely descriptive, as the comparison between the two interventions is a secondary outcome for which this study was not powered.

A post-hoc analysis of correlations of selected patient-reported outcome measures and gait analysis parameters is available in Supplemental File.

## Results

**Figure 3.**
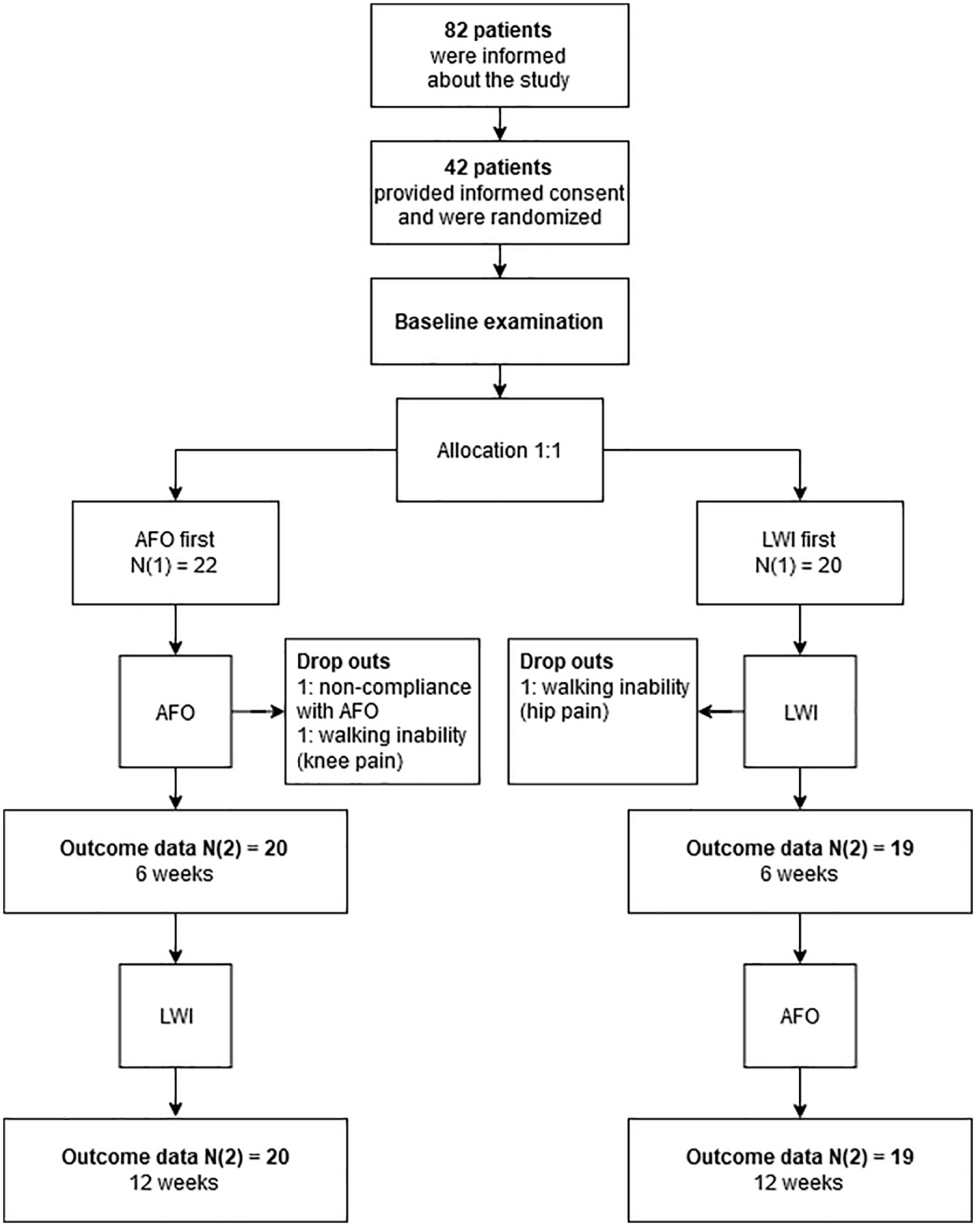
Patient flow in the study. AFO: ankle-foot orthosis; LWI: lateral wedge insole.

Forty-two participants met the inclusion criteria during the scheduled time frame and agreed to take part in the study after extensive information. Three patients dropped out of the study protocol. Figure 3 illustrates the flow of patients in the study. Table 1 illustrates baseline data of the remaining study cohort.

**Table 1.** Baseline characteristics of the study cohort.

Parameter	Cohort 1 mean [SD]	Cohort 2 mean [SD]	Total mean [SD]	*P*-value
Treatment sequence	LWI first	AFO first		
Age [years]	57.8 [7.9]	59.9 [9.0]	58.9 [8.4]	0.46
Height [cm]	173 [9]	172 [10]	172 [10]	0.73
Weight [kg]	86.9 [15.0]	88.1 [15.7]	87.6 [15.1]	0.81
BMI [kg/m²]	29.1 [4.6]	29.8 [4.4]	29.5 [4.5]	0.63
	Number [*N*]			
Male	9	11	20	0.64
Female	10	9	19	
K-L degree 1	6	3	9	0.33
K-L degree 2	7	9	16	
K-L degree 3	6	8	14	
Intervention side				
Right	8	9	17	0.96
Left	7	7	14	
Bilateral	4	4	8	
Total	19	20	39	

Anthropometric data, age, gender distribution and degrees of knee osteoarthritis according to Kellgren and Lawrence of the study cohort at baseline. The statistical comparisons revealed no significant differences between the cohorts.

AFO: ankle-foot orthosis; BMI: Body-Mass-Index; K-L degree: Kellgren and Lawrence degree of knee osteoarthritis on the analyzed side; LWI: lateral-wedge insoles; SD: standard deviation.

At baseline, the most common comorbidities were orthopedic disorders of the lower extremity: Contralateral knee pain (23 cases, including bilateral osteoarthritis), hip pain (13 cases) and foot deformities (11 cases). The most frequent foot deformities were pes planus, valgus and transversoplanus, often in combination. The frequency and type of foot deformities did not differ between the treatment sequence cohorts. In the standardized clinical examination, nine patients presented a knee extension deficit of three to five degrees.

Regarding the American Knee Society Clinical Rating System, almost half of the participants cited one-sided and one-third bilateral knee disease as the cause of their limitation when walking, while 12% (*N* = 5) were more severely restricted by other comorbidities.

After both interventions, patient-reported outcome parameters showed small significant improvements in EQ-5D Index, Oxford Knee Score, American Knee Society knee score and knee pain (Table 2). No significant difference between interventions was found.

**Table 2.** Statistical evaluation of patient-reported outcomes.

Test	Baseline [IQR]	LWI [IQR]	Median change	*P*-value LWI	AFO [IQR]	Median change	*P*-value AFO	*P*-value AFO versus LWI
FFbH-OA [%]	83 [72–92]	85 [75–97]	+3	0.12	86 [72–94]	±0	0.57	0.91
Oxford knee score	38 [32–41]	**39 [35–43]**	**+2**	**0.003**	**41 [34–43]**	**+3**	**0.001**	0.24
AKS knee score	66 [51–71]	**77 [61–86]**	**+6**	**0.01**	**80 [61–86]**	**+10**	**0.004**	0.29
AKS function Score	88 [70–100]	90 [75–100]	±0	0.07	90 [80–100]	±0	0.19	0.21
EQ-5D index	0.85 [0.68–0.90]	**0.88 [0.82–0.94]**	**+0.06**	**0.001**	**0.89 [0.78–0.94]**	**+0.04**	**0.002**	0.47
EQ VAS	75 [59–85]	80 [60–90]	±0	0.15	80 [65–90]	±0	0.07	0.69
Knee pain NRS	5 [3–7]	**3 [1–5]**	**−1**	**0.008**	**3 [2–5]**	**−1.5**	**0.004**	0.50

Patient-reported outcome parameters at baseline and after six weeks use of ankle-foot orthosis or lateral wedge insole, median and interquartile range (IQR). Median changes from baseline. Statistically significant changes are printed in **bold** letters.

FFbH-OA: Hannover Functional Ability Questionnaire-Osteoarthritis; AKS: American Knee Society Clinical Rating System; EQ-5D Index: European Quality of Life Group 5-Dimension Self-Report Questionnaire Index; NRS: numerical rating scale; VAS: visual analogue scale; LWI: scores after six weeks of lateral wedge insole use; AFO: scores after six weeks of ankle-foot orthosis use.

Baseline values of the EQ-5D Health State showed limitations in the categories pain/discomfort and less so in mobility and usual activities. All five dimensions shifted positively toward minor problems after intervention. This small change was significant only for “usual activities” and did not differ between interventions.

**Figure 4.**
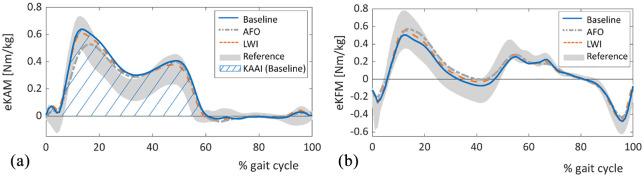
External knee torques of the patients with aids compared to the baseline and reference data from the same laboratory: (a) external knee adduction moment (eKAM) normalized to body weight, (b) external knee flexion moment (eKFM) normalized to body weight. Blue line – patients at baseline, gray line – patients with ankle-foot orthosis (AFO), orange line – patients with lateral wedge insoles (LWI), gray area – reference data ± standard deviation. Knee adduction angular impulse (KAAI) is the area under the curve of external knee adduction moment. The blue shaded area demonstrates knee adduction angular impulse during stance for the baseline condition.

In order to evaluate and compare biomechanical effects of the interventions, gait analysis was performed at baseline and with each orthopedic aid. Both interventions significantly reduced the first peak of the external knee adduction moment and the knee adduction angular impulse. These reductions were significantly greater with the ankle-foot orthosis than with the lateral wedge insole. In contrast to the orthosis, the lateral wedge insole also slightly reduced the second peak of the adduction moment. The ankle-foot orthosis, on the other hand, caused a 10% increase of the maximum knee flexion moment during stance. Changes in frontal and sagittal knee moments are shown in Figure 4.

Both orthopedic aids led to a small increase in step width and stance phase duration as shown in Table 3. Temporo-spatial parameters did not differ significantly between the two interventions.

**Table 3.** Gait analysis parameters.

Parameter	Baseline [SD]	AFO [SD]	*P*-value AFO	LWI [SD]	*P*-value LWI	*P*-value AFO vs. LWI
Speed [m/s]	1.30 [0.15]	1.30 [0.16]	0.82	1.31 [0.16]	0.59	0.83
Stride length [m]	1.42 [0.14]	1.43 [0.15]	0.41	1.44 [0.14]	0.22	0.80
Stance duration [%]	64.1 [1.7]	63.8 [1.8]	0.19	**63.6 [1.5]**	**0.03**	0.31
Single support [%]	35.6 [1.8]	35.5 [1.7]	0.63	35.6 [1.7]	0.90	0.65
Step width [cm]	8.5 [2.7]	**9.2 [2.5]**	**0.001**	**8.9 [2.4]**	**0.03**	0.14
GRF total 1st peak [N/kg]	11.2 [0.9]	11.4 [1.1]	0.15	11.3 [1.1]	0.26	0.27
GRF total 2nd peak [N/kg]	10.7 [0.7]	10.6 [0.7]	0.61	10.6 [0.8]	0.07	0.15
KAM 1st peak 0%–30% GC [Nm/kg]	0.67 [0.15]	**0.55 [0.19]**	**<0.001**	**0.63 [0.17]**	**0.02**	**0.001**
KAM 2nd peak 30%–60% GC [Nm/kg]	0.43 [0.13]	0.42 [0.14]	0.24	**0.41 [0.13]**	**0.03**	0.20
KFM peak [Nm/kg]	0.56 [0.23]	**0.61 [0.21**]	**0.05**	0.60 [0.23]	0.13	0.54
KAAI [Nm.s/kg]	21.1 [4.8]	**18.8 [5.9]**	**<0.001**	**20.1 [5.9]**	**0.05**	**0.02**

Participants’ gait analysis parameters for the analyzed limb, mean and standard deviation (SD).

Statistically significant changes are printed in **bold** letters.

AFO: gait analysis with ankle-foot orthosis; Baseline: gait analysis at baseline; GRF: ground reaction force; KAM: knee adduction moment; KAAI: knee adduction angular impulse; KFM: knee flexion moment; LWI: gait analysis with lateral wedge insoles; *P*-value AFO/*P*-value LWI: *P*-value of AFO or LWI gait analysis versus baseline.

The result of the post-hoc power analysis for the external knee adduction moment was 0.91.

## Discussion

The main finding of this study was the equal inability of both aids to improve patient-reported outcomes to a clinically relevant extent. Clinical osteoarthritis scores and health-related quality of life did not differ between the two interventions, so that no superiority of either aid could be determined. Regarding biomechanical effects, a significant difference between the aids was identified: Although both aids caused a reduction of the external knee adduction moment and its impulse, these reductions were larger with the ankle-foot orthosis than with the lateral wedge insole. The relatively small cohort size of 39 patients and the cross-over design require careful interpretation.

The patient-reported outcome measures showed a slight improvement by both aids, but the significance level was only achieved in knee pain, Oxford Knee Score, American Knee Society knee score and EQ-5D Index. No significant differences were found between the two aids. A significant superiority of one of the aids could therefore not be determined.

So far, only a few and partly controversial results have been obtained on ankle-foot orthoses in the context of knee osteoarthritis. Studies by Petersen et al.^21^ and Menger et al.^22^ reported positive influences on pain and functional outcomes., On the contrary, the study by Sliepen et al.^23^ described a clinically relevant deterioration of the same measures after a six-week intervention with an ankle-foot orthosis. Our results complement this spectrum: Health-related quality of life was improved by the ankle-foot orthosis and even by the lateral wedge insole despite the only marginal biomechanical effects of the latter. However, the changes in the monitored arthritis scores did not reach a clinically relevant extent.

Regarding temporo-spatial parameters in gait analysis, no relevant change could be determined with either of the two interventions. This finding confirms the results of Schmalz et al.^11^ and Mannisi et al.,^24^ who also found no differences in walking speed with ankle-foot orthosis or lateral wedge insole.

At the biomechanical level, the external knee adduction moment, which correlates with the severity of the varus deformity, represents a dynamic parameter for the load distribution of the knee joint in the frontal plane and is used as a surrogate parameter for the disease progression.^25^ Especially in the early stance phase, there is a close correlation between external knee adduction moment and the contact forces in the medial compartment of the knee.^5^ The reduction of external moments in the frontal plane is therefore the most important target in the treatment of medial knee osteoarthritis.^11^

Our results show a reduction of the first maximum of the external knee adduction moment by 18% with the ankle-foot orthosis and smaller effects on both maxima with the lateral wedge insole. The reduction of the first peak of the external knee adduction moment with the ankle-foot orthosis was significantly greater. Comparable results have already been shown by Schmalz et al.^11^ and Fantini Pagani et al.^7^ on healthy study groups. These effects were also confirmed in patients with knee osteoarthritis.^8,23^ Miyazaki described a 6.46 times higher risk of osteoarthritis progression with each 20% increase of overall peak external knee adduction moment.^6^ For this reason, the authors concluded that the 5%–10% reduction typically achieved with lateral wedge insoles may already be clinically meaningful. In this context, our results confirm previously known biomechanical ankle-foot orthosis effects, but with a lower effect size. The effect on the external knee adduction moment may have been reduced in our setting by individually adjusting the ankle-foot orthosis setting for the participants; in the aforementioned studies, measurements were taken in a maximum valgizing setting.

The biomechanical results of the lateral wedge insole confirm critical literature results from the past. Current meta-analyses and reviews describe small but significant positive influences on the biomechanical surrogates.^26–28^ The results of our study fit into this picture by showing a small effect of possible clinical relevance, as stated above.

The use of both aids also resulted in a significant reduction of the knee adduction angular impulse. This measure expresses the cumulative effect of the external knee adduction moment during the stance phase of gait. The reduction was significantly greater with the ankle-foot orthosis. The reduction of this parameter can be interpreted positively, because the knee adduction angular impulse provides a higher prediction accuracy regarding disease progression.^29,30^

In the sagittal plane, the external knee flexion moment is of major importance with regard to disease progression.^31^ Preliminary examinations described an unfavorable increase of up to 71% by ankle-foot orthosis.^8,32^ This effect was smaller in our collective. A possible reason is an overall weakened biomechanical effect of the ankle-foot orthosis due to the individual adaptation to the gait pattern of the patient without exhausting the maximum achievable effects.

The significant differences between the aids in the biomechanical surrogates were not reflected in the results of the patient-reported outcome measures. Despite discussion regarding biomechanical and clinical effects, 18 participants decided to continue using the ankle-foot orthosis at the end of the study. About 14 chose to continue using the lateral wedge insole. This suggests that there are factors beyond these parameters that influence the patient’s use of orthopedic aids.

When interpreting the results of this study, the relatively small number of cases must be considered. As the observed clinical effects were significant but rather small, we do not expect to find a relevant difference between the aids in a larger cohort. For the biomechanical parameters and especially the knee adduction moment, the result of the post-hoc power analysis supports this assumption. Additionally, our findings are concordant with previous literature.

The crossover study design did not have a washout phase between the two orthopedic aids. It cannot be ruled out that this may have a systematic influence, but we found no evidence of sequence-dependent carry-over effects in our statistical analysis.

In gait analysis, placement of the superior iliac spine marker was compromised in subjects with increased BMI (consult the supplement for more detail). Knee joint kinetics, however, are primarily influenced by the knee joint center position, knee axis rotation and the ground reaction force vector. Thus, no changes in knee joint kinetics are expected due to additional abdominal soft tissue.

In summary, this study shows that lateral wedge insoles and ankle-foot orthoses do not differ in their clinical improvements after six weeks of use. Biomechanically, the ankle-foot orthosis causes a significantly larger reduction of medial knee load than the lateral wedge insole. As this biomechanical difference was not reflected in patient-reported outcomes, the link between biomechanical and clinical changes produced by the aids remains unclear. A clinically relevant improvement of pain and functional outcomes cannot be generally assumed for all patients. This makes uncritical use of the devices questionable. Nevertheless, since the majority of patients experienced a pain reduction with both aids, we consider a therapy attempt justified in mild to moderate knee osteoarthritis.

The heterogeneous response to both devices highlights a need for research to determine patient characteristics that better predict which patients fully harness the benefits of the biomechanical changes. Regarding the promising biomechanical effects of the ankle-foot orthosis, insights on the long-term influence on disease progression would prove to be valuable.

**Clinical message**

- Both lateral wedge insoles and ankle-foot orthosis were associated with equal, slight improvements in knee pain and health-related quality of life in patients with medial knee osteoarthritis.
- Biomechanically, the ankle-foot orthosis causes a more reduction of medial knee load.

## Supplemental Material

sj-pdf-1-cre-10.1177_0269215521993636 – Supplemental material for A comparison between laterally wedged insoles and ankle-foot orthoses for the treatment of medial osteoarthritis of the knee: A randomized cross-over trial Click here for additional data file. Supplemental material, sj-pdf-1-cre-10.1177_0269215521993636 for A comparison between laterally wedged insoles and ankle-foot orthoses for the treatment of medial osteoarthritis of the knee: A randomized cross-over trial by Martin Schwarze, Leonie P Bartsch, Julia Block, Merkur Alimusaj, Ayham Jaber, Marcus Schiltenwolf and Sebastian I Wolf in Clinical Rehabilitation
