# Supplementary material for: A comparison between laterally wedged insoles and ankle-foot orthoses for the treatment of medial osteoarthritis of the knee: A randomized cross-over trial
Source: Clin Rehabil. 2021 Mar 29;35(7):1032–43. doi: 10.1177/0269215521993636 (PMC8193604; doi:10.1177/0269215521993636)
Supplement: sj-pdf-1-cre-10.1177_0269215521993636 – Supplemental material for A comparison between laterally wedged insoles and ankle-foot orthoses for the treatment of medial osteoarthritis of the knee: A randomized cross-over trial [file sj-pdf-1-cre-10.1177_0269215521993636.pdf]

Supplement

Gait analysis methodology

For the gait analysis, a 120 Hz 12-camera system (Vicon, Oxford, United Kingdom) and two force plates (Kistler, Winterthur, Switzerland) were used. Reflective markers were placed on bony landmarks according to PlugInGait (Vicon, Oxford, United Kingdom). The correct marker placement was checked independently by two examiners. A picture of each subject was saved for documentation.

When walking with the ankle-foot orthosis, no marker could be attached directly to the lateral malleolus on the affected side. The marker was placed on the orthosis instead. To compensate for the mismatch, a static calibration was carried out, in which the true position of the lateral malleolus was defined via the relative position to four additional cluster markers on the frontal tibia.[1]

In various subjects, the superior iliac spine marker could not be positioned directly on the bone due to increased soft tissue. The iliac spine was palpated, and the marker was displaced to the anterior, preserving the correct position in the frontal plane.

The patients repeatedly walked a distance of 7 m at a self-selected speed. The study participants carried out the gait analysis in their own footwear. The footwear was identical during all at all examination dates. At baseline, only shoes were worn. After each intervention period, the corresponding aid was worn in the gait analysis.

Motion data was recorded using the software Vicon Nexus 2.8 (Vicon, Oxford, United Kingdom). Gait events and force plate hits were set manually for one stride in each trial. In the ankle-foot orthosis trials, the respective lateral ankle marker was replaced by the position acquired in the static calibration trial, relative to the tibial markers, using a custom Matlab routine. Trials were filtered using the VCM Spline Filter in Vicon Nexus before applying the

PlugInGait model. Joint angles and moments in the sagittal, frontal and transverse plane as well as ground reaction forces and temporo-spatial parameters were then exported with a custom Matlab tool. All trials of each patient in each condition were graphed for one gait cycle to display joint angles in all 3 planes and ground reaction forces. For each condition, trials were checked visually for a consistent gait pattern. Trials with visible strong aberrations from this gait pattern were removed. Parameters were averaged over a minimum of 8 trials of each condition. The knee axis was defined with a knee alignment device set four times by two different investigators.

The evaluation of the gait analysis was focused on:

- External knee adduction moment (primary endpoint)
- Knee adduction angular impulse
- External knee flexion moment
- Temporo-spatial parameters

In an explorative approach, the average of all patients was graphed comparing the three conditions and the reference group. The data was screened visually for changes between conditions as well as for differences between patients and reference group. Parameters showing visible differences between conditions were further investigated.

Gait analysis limitations:

Placement of the superior iliac spine markers was compromised in subjects with soft tissue covering the pelvic bone, a problem reflecting the increased BMI of the study population. The resulting anterior displacement of the hip joint center must be addressed as an error affecting mostly sagittal kinematics. Knee joint kinetics, however, are primarily defined by the position of the knee joint center, the ground reaction force vector and knee axis rotation, all of which

were accurately assessed. Therefore, only the usual error margins for standardized 3D gait analysis apply to the main outcome measures.

1. Reulbach M. *Bestimmung des oberen Sprunggelenkzentrums für die instrumentelle 3D Bewegungsanalyse bei sprunggelenkübergreifenden Orthesen*. Bachelor's Thesis, Technische Hochschule Mittelhessen, 2019.
